# Complete Genome Sequence of GD1696, a Low-Virulence Strain of Human-Associated ST398 Methicillin-Susceptible Staphylococcus aureus

**DOI:** 10.1128/MRA.00688-19

**Published:** 2019-07-11

**Authors:** Jo-Ann McClure, Steven M. Shideler, Kunyan Zhang

**Affiliations:** a Centre for Antimicrobial Resistance, Alberta Health Services/Alberta Public Laboratories/University of Calgary, Calgary, Alberta, Canada; b Department of Pathology & Laboratory Medicine, University of Calgary, Calgary, Alberta, Canada; c Department of Microbiology, Immunology and Infectious Diseases, University of Calgary, Calgary, Alberta, Canada; d Department of Medicine, University of Calgary, Calgary, Alberta, Canada; e The Calvin, Phoebe and Joan Snyder Institute for Chronic Diseases, University of Calgary, Calgary, Alberta, Canada; Georgia Institute of Technology

## Abstract

The emerging livestock-associated Staphylococcus aureus multilocus sequence type 398 (ST398) appears to have augmented virulence in humans. However, it is unclear if all ST398 strains are equally virulent. Here, we present the chromosomal sequence of a low-virulence ST398 methicillin-susceptible S. aureus (MSSA) strain, GD1696, to investigate ST398 sublineage virulence.

## ANNOUNCEMENT

An increasing number of severe infections caused by emerging livestock-associated Staphylococcus aureus multilocus sequence type 398 (ST398) strains have been observed. Initial cases were reported in patients working in close contact with farm animals, but the strain has gone on to cause infection in patients lacking contact with livestock (1–10). While there appears to be augmented pathogenicity of ST398 methicillin-susceptible S. aureus (MSSA), specifically in humans, it has not been determined if all ST398 strains are equally virulent. To that end, we used the Caenorhabditis elegans infection model to test a collection of ST398 MSSA strains and found that they could be clustered into high-, moderate-, and low-virulence groups, with mean nematode killing rates of 90%, 67%, and 44%, respectively. Whole-genome sequencing was used to elucidate virulence factors that could be responsible for the various toxicities. In separate reports, we presented the full chromosomal sequence of a high-virulence strain, GD487, as well that of a moderate-virulence strain, GD1108. This report presents the complete genome sequence of a low-virulence ST398 MSSA strain, GD1696.

Strain GD1696 was obtained from an inpatient from a prevalence survey in 2011 in Guangzhou, People’s Republic of China. A single colony was picked from a tryptic soy agar plate and grown overnight in liquid culture at 37°C, at which time genomic DNA was isolated using phenol-chloroform extraction. Library preparation and DNA sequencing were performed at the Genome Quebec Innovation Centre in Montreal, Canada. Sequencing using PacBio RS II technology and a single-molecule real-time (SMRT) cell was done on a sheared large-insert library that was generated with Covaris g-TUBEs and the SMRTbell template prep kit 1.0. An Illumina library was prepared with the Nextera XT library preparation kit, and sequencing, with a 600-cycle MiSeq v3 instrument, was performed at the Nicole Perkins Microbial Communities Core Laboratory at the University of Calgary in Canada. Using default settings for all software, sequence quality was assessed with FastQC v0.11.5 (http://www.bioinformatics.babraham.ac.uk/projects/fastqc), and trimmed Illumina reads (including removal of sequences with a quality score of <20) were generated with Cutadapt v1.15 (11). Hybrid sequence assembly was performed with the Unicycler v0.4.7 pipeline (SPAdes v3.13.0, minimap, Racon v1.3.2, and Pilon v.1.23) (12–16). GC content of the assembled product was determined with QUAST v4.4 (17). Genes were annotated using the NCBI Prokaryotic Genome Annotation Pipeline using the best-placed reference protein set (GeneMarkS-2+ v4.8) (18).

Hybrid assembly of the trimmed Illumina and filtered PacBio reads produced one contig, representing the chromosome. There were 305,952 Illumina reads, with average read lengths of 226 bp for R1 and 210 bp for R2 and an estimated genome coverage of 24×. There were 98,382 raw PacBio reads covering 897,403,420 sequenced bases, with an average read length of 9,121 bp and an estimated genome coverage of 284×. The resulting chromosome was 2,801,264 bp long with a GC content of 32.94%. Following annotation, 2,819 genes were identified, of which 2,741 were coding sequences (CDS), 78 were RNA genes, and 85 were pseudogenes.

Detailed analysis of the high-, moderate-, and low-virulence genomes is under way to explore potential insights into ST398 sublineage virulence.

### Data availability.

The genome sequence was deposited at GenBank under the accession number CP040233 and SRA accession numbers SRX5923213 (Illumina) and SRX5923212 (PacBio).
